# The Chemical Composition and Nematicidal Activity of Wasabi (*Eutrema japonicum*) Rhizome Extract Against *Meloidogyne enterolobii*

**DOI:** 10.3390/plants14213310

**Published:** 2025-10-30

**Authors:** Jiali Wang, Zhiwen Li, Ying Wei, Jiguang Luo, Xiaoli Dou, Meiying Fu, Xiangping Zeng, Bao Wang, Zhixiang Zhao, Huifang Wang, Baibi Zhu

**Affiliations:** 1 Key Laboratory of Plant Diseases and Pests of Hainan Province, Institute of Plant Protection (Research Center of Quality Safety and Standards for Agro-Products, Hainan Academy of Agricultural Sciences), Hainan Academy of Agricultural Sciences, Haikou 571100, China; wjialihainan@163.com (J.W.); lizhiwen2017@126.com (Z.L.); nkywy0909@163.com (Y.W.); luojiguang@hnaas.org.cn (J.L.); dou1653357612@163.com (X.D.); fumeiying@hnaas.org.cn (M.F.); zengxiangping@hnaas.org.cn (X.Z.); wangbao@hnaas.org.cn (B.W.); zhaozhixiang@hnaas.org.cn (Z.Z.); 2 Institute of Vegetables, Hainan Academy of Agricultural Sciences, Haikou 571100, China

**Keywords:** *Eutrema japonicum*, *Meloidogyne enterolobii*, sec-butyl isothiocyanate, geraniol, biocontrol, network pharmacology analysis

## Abstract

*Eutrema japonicum* is a perennial herb belonging to the *Eutrema* genus in the crucifer family. In recent years, numerous substances with notable pharmacological activities have been successfully isolated from *E. japonicum*. Despite significant advancements in related research, the efficacy of the rhizome extract of *E. japonicum* against root-knot nematodes remains unknown. In this study, the rhizome extract of *E. japonicum* was used as raw material to demonstrate the inhibitory and nematicidal effects of the extract on *Meloidogyne enterolobii*. The results showed that the LC_50_ of the *E. japonicum* rhizome extract on second-stage juveniles (J2s) was 69.590 mg/mL and 22.336 mg/mL at 24 h and 48 h after treatment, respectively. The mortality rate of J2s reached 88.93% at 48 h post-treatment when the concentration was 200 mg/mL, and the inhibition rate of single-egg hatching reached 88.14%. This study analyzed the chemical composition of the ethanol extract of *E. japonicum*, and 10 organosulfur compounds and lipid compounds with insecticidal and antibacterial effects were preliminarily screened out. Among them, sec-butyl isothiocyanate and geraniol were further investigated for their nematicidal activity, demonstrating high efficacy against *M. enterolobii*. Moreover, we conducted network pharmacology analysis and RT-qPCR analysis to predict the potential inhibitory mechanisms of sec-butyl isothiocyanate and geraniol on *M. enterolobii*. These findings offer a scientific foundation and theoretical framework for utilizing *E.japonicum* as a potential raw material for developing novel natural plant nematicides.

## 1. Introduction

*Meloidogyne enterolobii* is recognized as one of the most destructive root-knot nematodes, with a wide host range, which infects most crops, including monocotyledonous, dicotyledonous herbaceous, and woody crops [1,2]. They pose particularly severe threats to cucurbit, leguminous, and solanaceous crops [3]. What is more, it can parasitize crops carrying resistant genes that are effective against other nematodes, such as the *Mi-1* gene in tomatoes, the *N* gene in peppers, and the *Rk* gene in cowpea, leading to substantial yield losses that can exceed 65% [4]. *M. enterolobii* is widely distributed in tropical and subtropical regions, especially in Hainan Province, China, which has a tropical climate conducive to the rapid spread of nematode populations without overwintering, thus facilitating the occurrence and dissemination of *M. enterolobii*. Huang et al. focused on characterizing root-knot nematode populations in cucurbit vegetables on Hainan Island, revealing a high incidence of *M. enterolobii*, reaching up to an 80% incidence rate [5]. Li et al. reported a detection rate of over 60% for *M. enterolobii* on vegetables in Hainan Province [6]. This finding suggests that *M. enterolobii* has replaced *M. incognita* as the primary pathogenic nematode species impacting vegetables in the region. Consequently, the prevention and control of *M. enterolobii* are crucial for agricultural production in Hainan Province, China [6,7].

Currently, chemical methods are predominantly utilized for the management of root-knot nematodes. Chemical agents are categorized into fumigants (mainly halohydrocarbons and isothiocyanates) and non-fumigants (mainly organophosphates and carbamates) [8]. Fumigants kill nematodes via volatile gases or liquids released in soil, and commonly used fumigants such as metam sodium exhibit good control effects, as they generate methyl isothiocyanate in soil to exert nematicidal and bactericidal actions [2]. Abamectin is the most widely used non-fumigant, but it is easily degraded by soil microorganisms and has a short persistence, thus its being often combined with other agents in practical production to enhance nematicidal efficacy [9]. However, the problems of resistance and residues in traditional chemical control agents pose significant challenges. Since the 1980s, there has been a growing interest in the use of plant extracts for controlling root-knot nematodes [10]. Previous studies have shown that root extracts of plants such as *Chromolaena odorata* and *Cymbopogon citratus* exhibit strong inhibitory and nematicidal activities against soybean root-knot nematodes [11]. In addition, ethanol extracts from 22 plant species, including *Glycyrrhiza uralensis*, *Dictamnus dasycarpus*, *Scutellaria baicalensis*, and *Gentiana scabra*, exhibit certain nematicidal activities against both second-stage juveniles (J2s) and egg masses of cucumber root-knot nematodes, with the nematicidal activity enhancing as the treatment time is prolonged [12]. Nematicidal compounds derived from natural plants offer advantages such as biodegradability in soil, beneficial effects on biological health, and environmental safety, making them a focal point in biopesticide research [13,14,15].

*Eutrema japonicum*, commonly known as wasabi, is a perennial herbaceous plant belonging to the Cruciferae family. It prefers cold and humid weather and is native to Japan. In China, it is mainly planted in some mountainous areas such as Yunnan and the western and southwestern regions of Sichuan [16]. *E. japonicum* is often referred to as “green gold” due to its green exterior; it is chiefly valued for the distinctive pungent flavor it develops when crushed and is mainly consumed for this characteristic taste, and its rhizomes are widely used as a condiment in various cuisines [17]. *Eutrema japonicum* is recognized as a valuable medicinal plant with a range of pharmacological benefits attributed to its high concentration of isothiocyanate compounds [18,19]. Studies have demonstrated that these isothiocyanates, responsible for wasabi’s spiciness, exhibit nematicidal activity [20]. Moreover, wasabi essential oil has very good antibacterial activity against phytopathogenic fungi (such as *Aspergillus* and *Penicillium*) [18]. It can also be used as an antibacterial agent, insecticide, and herbicide [21]. Owing to its notable pharmacological profile, *E. japonicum* represents a promising herbal medicine worthy of further investigation and development.

This study aims to evaluate the inhibitory activity of an ethanol extract derived from *E. japonicum* rhizomes against *M. enterolobii* and to conduct a preliminary screening of its potential active constituents. Furthermore, selected compounds with nematicidal potential are assessed for their in vitro activity. The findings are expected to provide a scientific foundation and theoretical reference for utilizing *E. japonicum* as a source material in developing novel natural nematicides.

Network pharmacology is a comprehensive discipline combining systems biology and bioinformatics that plays an important role in the study of multi-component and multi-target mechanisms of traditional Chinese medicine (TCM) [22]. In recent years, network pharmacology has also been gradually applied in the agriculture field. For example, it has been used to study the toxicity mechanism of pesticides to humans and to screen plant-derived components that can evade the quarantine pest *Zeugodacus cucurbitae* (Coquillett) [23,24,25]. Such approaches can accelerate the screening of natural compounds extracted from plants and the development of botanical pesticides [26].

This study investigates the inhibitory effect of an ethanol extract from *E. japonicum* rhizome against *M. enterolobii*, with a preliminary screening to identify potential active compounds. Selected candidates with nematicidal potential were further evaluated under controlled laboratory conditions. Using network pharmacology approaches, this study predicts potential targets and molecular mechanisms through which the extract inhibits *M. enterolobii*, followed by a preliminary validation using RT-qPCR. The findings aim to provide a scientific foundation and theoretical support for the development of *E. japonicum*-based natural nematicides.

## 2. Results

### 2.1. Effects of Different Co-Solvents on E. japonicum Rhizome Extract

In order to select an appropriate co-solvent for *E. japonicum* rhizome extract, three reagents were tested. The results indicated that dimethyl sulfoxide (DMSO) exhibited the most effective solubilization capacity after the extract was maintained in a clear solution without any precipitation throughout the 24 h and 48 h observation periods. Moreover, no nematode mortality was observed with DMSO treatment. By contrast, 1% aqueous acetone led to precipitate formation within 24 h and resulted in nematode death. Using sterile water for extract dissolution resulted in partial insolubility and precipitation at 24 h post-treatment. Therefore, 1% aqueous DMSO was selected for solubilizing the extract (Table 1).

### 2.2. Nematicidal Effect of E. japonicum Rhizome Extract

**Figure 1:**
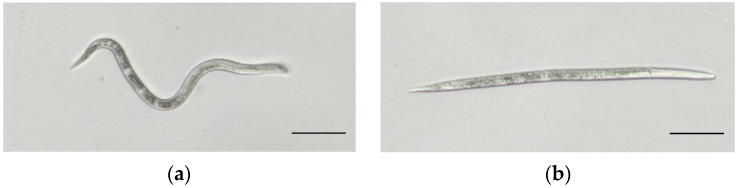
Second-stage juveniles (J2s) of *M. enterolobii*. Note: (**a**) Healthy J2. (**b**) Dead J2 after being treated with *E. japonicum* rhizome extract. Scale bars = 50 μm.

The results of the bioactivity assay indicated that the ethanol extract obtained from *E. japonicum* rhizome displayed significant nematicidal activity against J2s) of *M. enterolobii* (Figure 1). This bioactivity was significantly higher than that observed in the blank control group (sterile water) and solvent control (1% aqueous DMSO). Additionally, the bioactivity exhibited a tendency to increase with time. The LC_50_ values were determined to be 69.59 mg/mL and 22.336 mg/mL after 24 h and 48 h, respectively. Moreover, at a concentration of 200 mg/mL of the *E. japonicum* rhizome extract, the nematode mortality rate reached 88.93% after 48 h (Table 2 and Table 3).

### 2.3. Effects of E. japonicum Rhizome Extract on the Hatching of Nematode Single Egg

**Figure 2:**
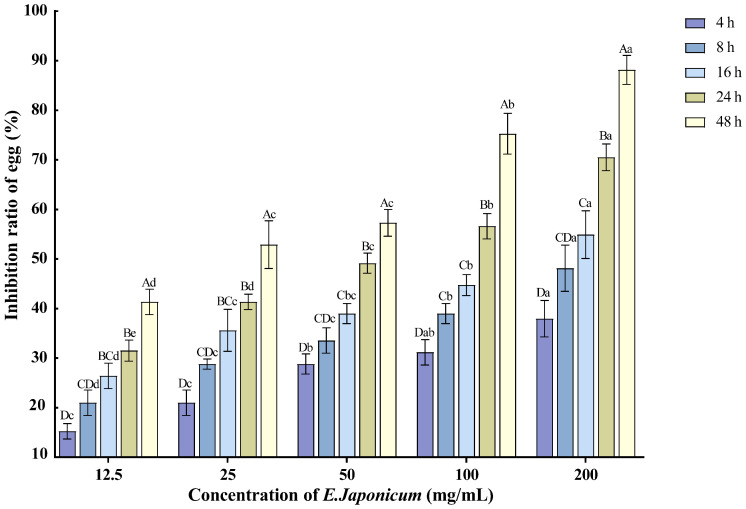
Effects of *E. japonicum* rhizome extract on the hatching of nematode single egg. Note: Bars marked with different capital letters between different times at the same concentration indicate significant differences (*p* < 0.05) as tested by Tukey’s method; bars marked with different lowercase letters between different concentrations at the same time indicate significant differences (*p* < 0.05) as tested by Tukey’s method.

The ethanol extract of the rhizome of *E. japonicum* at various concentrations exhibited inhibitory effects on the hatching of nematode single eggs of *M. enterolobii*. As the treatment duration extended and the concentration increased, the inhibition rate of egg hatching also rose. The 200 mg/mL concentration showed the most significant effect on nematode egg hatching, with the inhibition rate reaching 88.14% after 48 h of treatment (Figure 2).

### 2.4. Chemical Composition Analysis of the Rhizome Extract of E. japonicum

GC-MS-VOCs analysis yielded 1210 peaks. Through a review of the literature and other relevant studies, 10 organosulfur compounds, lipids, organic heterocyclic compounds, and other substances with insecticidal and bacteriostatic effects were initially screened from the extract (Table 4).

### 2.5. Activity Assay Results of Sec-Butyl Isothiocyanate from E. japonicum Rhizome Extract Against J2s of M. enterolobii and Single-Egg Hatching

#### 2.5.1. Activity of Sec-Butyl Isothiocyanate Against J2s of *M. enterolobii*

The outcomes of the activity assay indicated that the individual component, sec-butyl isothiocyanate, exhibited substantial biological activity against J2s of *M. enterolobii*, surpassing that of the negative control (sterile water). Additionally, the inhibitory effect showed a tendency to escalate over time. The LC_50_ values were 1.087 and 0.04 µg/mL after 24 h and 48 h of treatment, respectively. Moreover, the mortality rate of the nematodes reached 100% after 48 h when exposed to 10 µg/mL of sec-butyl isothiocyanate (Table 5 and Table 6).

#### 2.5.2. Effects of Sec-Butyl Isothiocyanate on the Hatching of Nematode Single Egg

**Figure 3:**
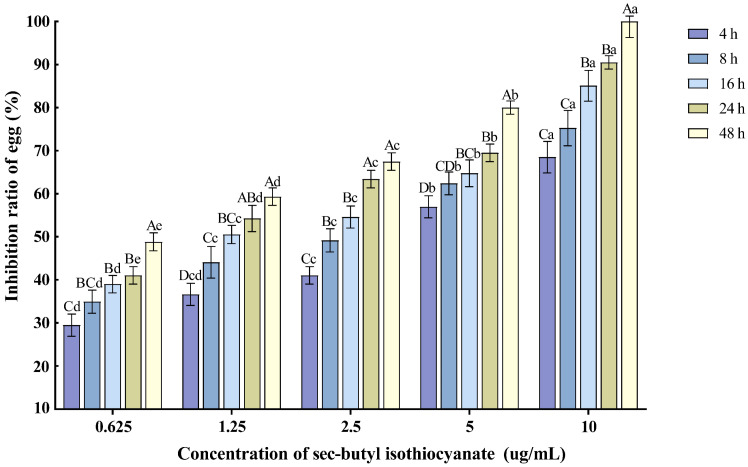
Effects of sec-butyl isothiocyanate on the hatching of nematode single egg. Note: Mean values marked with different capital letters between different times at the same concentration indicate significant differences (*p* < 0.05) as tested by Tukey’s method; mean values marked with different lowercase letters between different concentrations at the same time indicate significant differences (*p* < 0.05) as tested by Tukey’s method.

Various concentrations of sec-butyl isothiocyanate exhibited inhibitory effects on the hatching of *M*. *enterolobii* nematode eggs. As the treatment time extended and the concentration increased, the egg hatching inhibition rate also rose. Specifically, the 10 µg/mL concentration had the most significant impact on nematode egg hatching, with the inhibition rate reaching 90.51% after 24 h of treatment (Figure 3).

### 2.6. Activity Assay Results of Geraniol from E. japonicum Rhizome Extract Against J2s of M. enterolobii and Single-Egg Hatching

#### 2.6.1. Activity of Geraniol Against J2s of *M. enterolobii*

The bioassay results demonstrated that the individual compound, geraniol, exhibited significant nematicidal activity against J2s of *M. enterolobii*, outperforming the negative control (sterile water). Additionally, the inhibitory effect showed a tendency to escalate over time. The LC_50_ values were 1.018 and 0.062 µg/mL after 24 h and 48 h of treatment, respectively. Moreover, the mortality rate of the nematodes reached 100% after 48 h when exposed to 5 µg/mL of geraniol (Table 7 and Table 8).

#### 2.6.2. Effects of Geraniol on the Hatching of Nematode Single Egg

**Figure 4:**
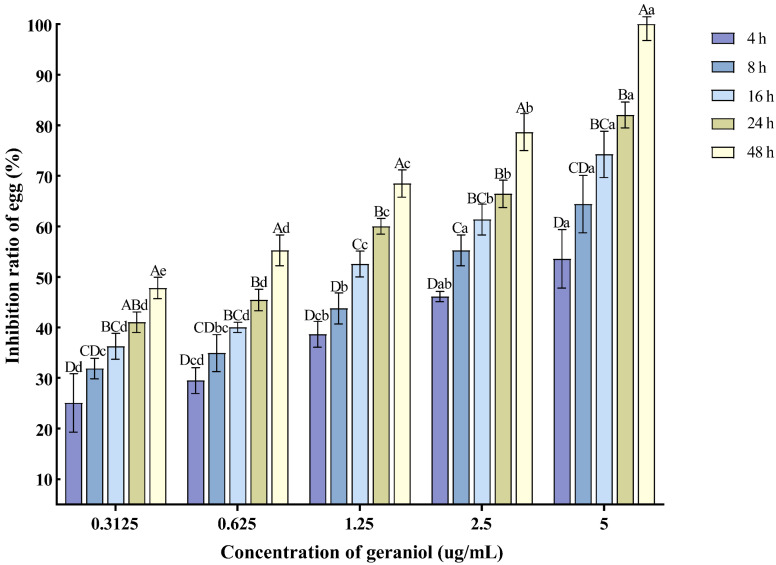
Effects of geraniol on the hatching of nematode single egg. Note: The mean values marked with different capital letters between different times at the same concentration indicate significant differences (*p* < 0.05) as tested by Tukey’s method; mean values marked with different lowercase letters between different concentrations at the same time indicate significant differences (*p* < 0.05) as tested by Tukey’s method.

With the extension of the treatment time and the increase in concentration, the inhibition rate of egg hatching also increased. The 5 μg/mL concentration exhibited the best inhibitory effect on nematode egg hatching, with the inhibition rate reaching 82.04% at 24 h post-treatment (Figure 4).

### 2.7. Results of Network Pharmacology Analysis

#### 2.7.1. Collection of Potential Targets for Active Compounds

Ten compounds were searched for their targets in the TCMSP database, and potential target association information was retrieved from the TCMID database. Functional annotation data of the targets were then obtained from the BATMAN-TCM database. SMILES sequences corresponding to the active ingredients were downloaded from the PubChem database and input into the Swiss Target Prediction database. After removing targets with reliability less than 50% and duplicates, a total of 1265 potential targets were obtained (Supplementary Table S1). The number of targets for each of the 10 compounds is as follows: 2-Butenal,(E)- (98); Butane,1-isothiocyanato- (154); Butane,2-isothiocyanato- (151); Acetaldehyde, tetramer (118); Furan,tetrahydro-2,2,4,4-tetramethyl- (172); geraniol (97); Isopropyl isothiocyanate (159); 2(4H)-Benzofuranone,5,6,7,7a-tetrahydro-4,4,7atrimethyl-,(R)- (114); and 2(5H)-Furanone (96), Pyrazine,tetramethyl- (106).

#### 2.7.2. Targets Collection of Root-Knot Nematode

Target screening of root-knot nematodes: Based on information from the academic databases GeneCards, OMIM, TTD, CTD, DisGeNET, and NCBI, potential targets of root-knot nematodes were screened. All identified targets were then consolidated, and duplicates were eliminated, resulting in a total of 503 specific targets for root-knot nematodes (Supplementary Table S2).

#### 2.7.3. The PPI Network of Common Targets Between Compounds and Root-Knot Nematodes

**Figure 5:**
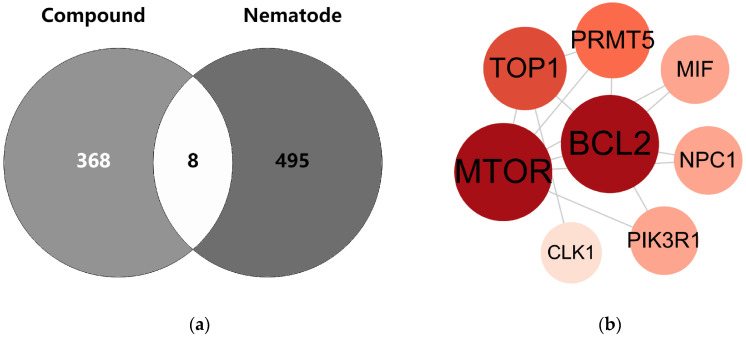
Venn diagram and PPI network of common targets of compounds and nematodes. Note: (**a**) Venn diagram: Light gray and dark gray represent the compound targets and nematode targets, respectively. The overlapping area indicates the common protein targets. (**b**) PPI network: Circular nodes with different colors and sizes represent various proteins; the lines between nodes denote the interaction relationships among these proteins.

The intersection between the compounds and targets of root-knot nematodes is depicted using a Venn diagram (Figure 5a). The eight intersecting targets identified in the figure were input into the STRING database to construct a PPI network. The network comprises 8 nodes and 13 edges. These nodes are mTOR, BCL2, TOP1, PRMT5, MIF, NPC1, PIK3R1, and CLK1 (Figure 5b).

#### 2.7.4. GO and KEGG Pathway Enrichment

**Figure 6:**
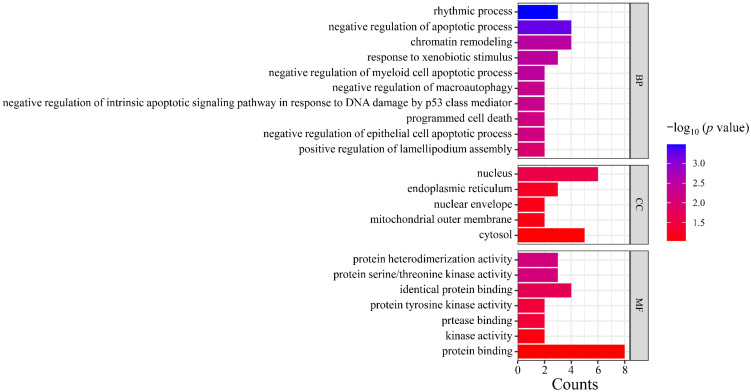
GO functional enrichment analysis. Note: GO, Gene Ontology; CC, cell components; MF, molecular function; BP, biological process.

**Figure 7:**
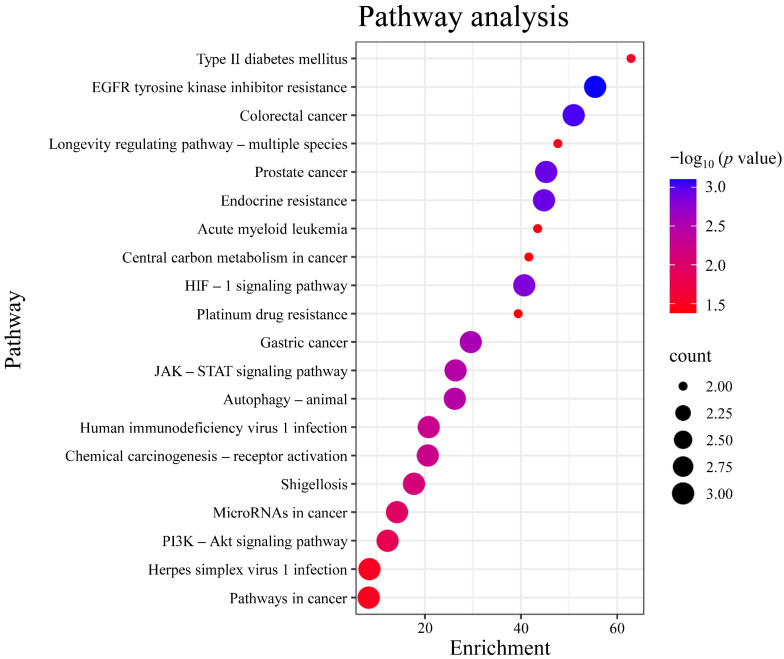
Analysis of KEGG pathway enrichment. Note: The color of each bubble corresponds to −log_10_ (*p*-value) (darker blue denotes higher statistical significance), while the bubble size is proportional to the count of genes enriched in each pathway. KEGG, Kyoto Encyclopedia of Genes and Genomes.

Gene Ontology (GO) analysis was performed on the targets in the PPI network using DAVID. The results indicated that the numbers of terms related to cellular component (CC), molecular function (MF), and biological process (BP) were 26, 18, and 24, respectively (*p* < 0.05). Based on the p value, the top three CCs were the nucleus, endoplasmic reticulum, and nuclear envelope, which are highly correlated with the cell nucleus and cytoplasm. The top three MFs were protein serine/threonine kinase activity, identical protein binding, protease binding, and protein tyrosine kinase activity. The top three BPs were chromatin remodeling, response to xenobiotic stimulus, and negative regulation of myeloid cell apoptotic process, which are closely related to the defensive response (Figure 6). After Kyoto Encyclopedia of Genes and Genomes (KEGG) signaling pathway analysis, a total of 20 signaling pathways were obtained (*p* < 0.05). The top five signaling pathways were pathways in cancer, herpes simplex virus 1 infection, PI3K-Akt signaling pathway, microRNAs in cancer, and shigellosis (Figure 7).

#### 2.7.5. Compound–Target Protein Interaction Network

**Figure 8:**
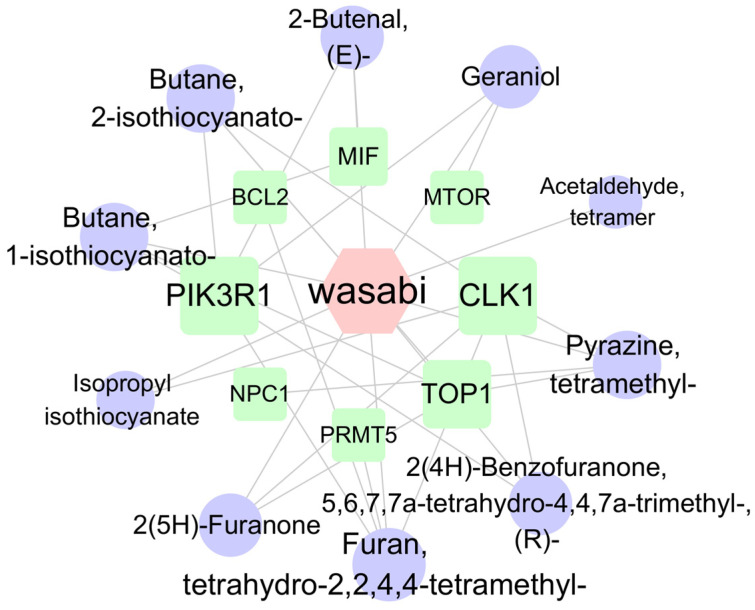
The network of compound–target protein interaction. Note: Circles represent wasabi compounds, squares represent targets, and hexagons represent wasabi. Nodes are connected by lines, with shorter distances indicating closer interaction relationships.

Cytoscape (version 3.10.2) was used to visualize the target proteins corresponding to the 20 pathways with the lowest *p*-values, resulting in a network diagram comprising 18 nodes and 32 edges (Figure 8). The target network diagram reveals that the target protein most closely associated with sec-butyl isothiocyanate is PIK3R1, while the target protein most closely related to geraniol is mTOR. Based on the names of the target proteins, a search was conducted in the root-knot nematode target database, yielding a total of 26 PIK3R1 homologous proteins and 12 rapamycin-insensitive companion of mTOR N-terminal domain-containing proteins (Supplementary Table S3). Subsequently, based on the LOC numbers corresponding to these proteins, a search was carried out in the NCBI database to obtain the corresponding protein sequences. The NCBI Conserved Domain Search [https://www.ncbi.nlm.nih.gov/Structure/cdd/wrpsb.cgi (accessed on 13 March 2025)] was utilized to analyze the protein structures. Finally, two PIK3R1-1 and PIK3R1-2 proteins that contain the SH2 conserved domain and two Rictor-1 and Rictor-2 proteins that contain the RICTOR_N conserved domain were identified.

### 2.8. Expression Patterns of PIK3R1 and Rictor Genes

**Figure 9:**
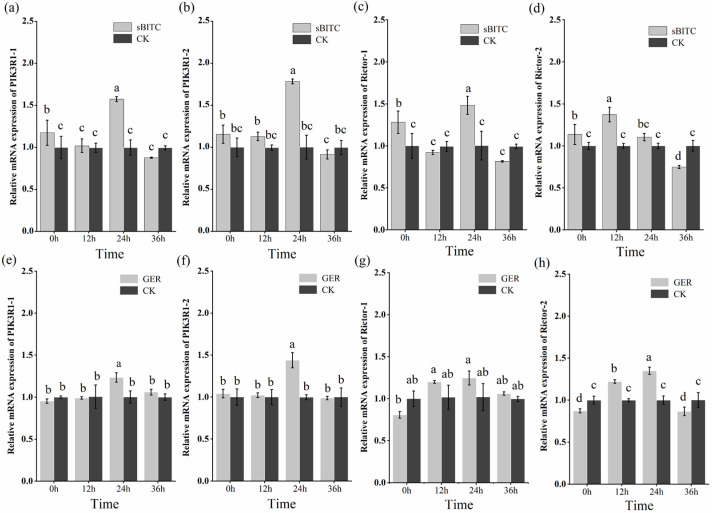
Analysis of gene expression patterns. Note: (**a**–**d**) Expression patterns of *PIK3R1* and *Rictor* genes in second-stage juveniles (J2s) after treatment with 1.25 μg/mL sec-butyl isothiocyanate (sBITC). (**e**–**h**) Expression levels of *PIK3R1* and *Rictor* genes in second-stage juveniles (J2s) after treatment with 1.25 μg/mL geraniol (GER). Columns marked with the different letters indicate significant differences (*p* < 0.05) as tested by Tukey’s method.

The J2s of *M. enterolobii* were treated with 1.25 μg/mL of sec-butyl isothiocyanate and 1.25 μg/mL of geraniol. The expression patterns of *PIK3R1* and *Rictor* were detected by RT-qPCR at 12 h, 24 h, and 36 h (Figure 9). The results indicate that sec-butyl isothiocyanate and geraniol were capable of enhancing the expression of *PIK3R1* and *Rictor* in J2s. In particular, after sec-butyl isothiocyanate treatment, compared with the control group, it was found that the *PIK3R1* gene and the *Rictor* gene showed a significant upward expression at 0 h. Among them, the expression level of *Rictor-2* peaked at 12 h post-treatment, while the expression levels of *PIK3R1-1*, *PIK3R1-2*, and *Rictor-1* reached their highest level 24 h post-treatment. At 36 h post-treatment, all four genes exhibited a significant decrease in expression, with levels significantly lower than those in the control group. After geraniol treatment, compared with the control group, *PIK3R1-1* and *PIK3R1-2* showed an upward expression trend, peaking at 24 h after treatment. Their expression levels subsequently decreased, with no significant difference compared to those at 0 h post-treatment. The key difference was that, compared with the control group, the expression levels of *Rictor-1* and *Rictor-2* first showed a downward trend at 0 h, then increased to a peak at 24 h, and then decreased again, but no significant difference in expression levels was observed between the 36 h group and 0 h group.

## 3. Discussion

Extracts from different wasabi varieties have been shown to possess insecticidal activity [20,35,36]; notably, fresh residue of *Wasabia japonica* exhibits inhibitory effects on the infection of *Meloidogyne incognita* (*M. incognita*) [20]. This study is the first to demonstrate that the ethanol extract of *E. japonicum* rhizome, along with its active compounds (sec-butyl isothiocyanate and geraniol), exhibits good biological activity against J2s and the single-egg hatching of *M. enterolobii*. This finding thereby contributes to the development of phytogenic nematicidal substances derived from *E. japonicum*.

The eggs and J2s of nematodes are key targets for control, as they occur in the rhizosphere and are easily controllable before infecting host roots [37,38]. This study showed that at 200 mg/mL, nematode mortality reached 88.93% after 48 h treatment, significantly higher than that of the chemical control (0.1 mg/mL abamectin, 68.33%). In addition, the 200 mg/mL concentration exerted the most significant inhibitory effect on nematode egg hatching, with an inhibition rate of 88.14% at 48 h post-treatment. Specifically, nematode mortality was directly correlated with the extract concentration and exposure time, consistent with the findings of Muhammad Ismail et al. [39]. By contrast, the solvent control (1% DMSO) resulted in a nematode mortality of 1.33% at 48 h, showing no significant difference from the negative control (sterile water) (Table 2). Because DMSO concentration was controlled below 0.5% in subsequent experiments, it was not used as a control in the subsequent experiment.

Information on the nematocidal constituents and action mechanisms of the ethanol extract from *E. japonicum* rhizome is of practical significance for controlling nematodes [40,41]. In the current investigation, chemical composition analysis via GC-MS-VOCs identified a total of 1210 peaks in the ethanol extract of *E. japonicum* rhizome. Furthermore, 10 of these compounds with insecticidal and bacteriostatic activities were organized and analyzed, including lipids, organic heterocyclic compounds, and other substances [30,31,32,33,34]. In recent years, numerous plant-derived monomeric constituents have been demonstrated to possess significant nematicidal properties, such as alkaloids [42], fatty acids [43], alcohols [44], aldehydes [45], terpenoids [46], and others. Additionally, studies have reported that isothiocyanate compounds can inhibit the motility of J2s and the single-egg hatching of *M. incognita* [28,29,30]. Geraniol, a monoterpene compound, has also been demonstrated to exhibit significant nematicidal activity against *Caenorhabditis elegans* [32]. In this study, treatment with 10 µg/mL sec-butyl isothiocyanate and 5 µg/mL geraniol resulted in egg hatching inhibition rates of 95.51% and 82.04%, respectively, after 24 h. After 48 h of treatment, the mortality rate of J2s reached 100% in both groups, which was significantly higher than that of the chemical control (100 µg/mL abamectin). According to the literature, isothiocyanates exhibit nematicidal activity: 60 μM benzyl isothiocyanate inhibited *Heterodera glycines* egg hatching (15.6% on day 14 after 4 h incubation), and this impact on incubation is rapid and long-lasting [47]; three isothiocyanates caused the 100% mortality of *Meloidogyne javanica* (*M. javanica*) J2s after 3 days [48]; and allyl isothiocyanate induced paralysis in *M. incognita* J2s [49]. Compound activity against nematodes varies by population: geraniol showed an EC_50_ of 430 µg/mL against pinewood nematodes [50]; 50 µg/mL geraniol completely inactivated *M. javanica* J2s and inhibited 70% of egg hatching [51]; and 50 µg/mL geraniol caused the 95.00% mortality of *Meloidogyne graminicola* J2s after 24 h [52]. Given that volatile substances degrade easily in the environment, field experiments are more significant than laboratory tests. Field trials showed that 1.0 kg/ha allyl or acetyl isothiocyanate controlled *M. javanica* better than metam sodium [48]; pot experiments found that sublethal geraniol reduced female *M. javanica* in tomato roots [51]. The results of this study further indicated that compounds from *E. japonicum* rhizome have good inhibitory effects on *M. enterolobii*. However, this study only investigated in vitro nematicidal activity. Subsequent studies need to further clarify the practical efficacy of the compounds against *M. enterolobii* under field conditions, combinations with other compounds that can exert a synergistic effect, and which adjuvants can delay the degradation rate of volatile compounds.

To explore the mechanism of *E. japonicum* compounds, this study combined network pharmacology and RT-qPCR to analyze their effects on nematode target proteins. Eight candidate targets were identified; among them, sec-butyl isothiocyanate and geraniol were the most strongly associated with PIK3R1 and mTOR, respectively, both closely linked to the PI3K-Akt pathway [53]. This is consistent with the report by Veeran et al. (2019) [54], in which curcumin was shown to induce autophagy and inhibit growth in insect *Spodoptera frugiperda* via suppression of the PI3K-Akt pathway. This suggests that the PI3K-Akt pathway plays a key regulatory role in the insecticidal mechanism of the active compounds [55]. According to the literature, the PI3K-Akt pathway is considered to be the main signaling pathway for defense responses in *M. incognita* when it infects soybean [56]. The PI3K-Akt pathway is associated with developmental processes and various cellular functions; during the early stages of parasitism of *M. incognita* by *Pasteuria penetrans*, the PI3K-Akt pathway was detected to be in an active state [57]. Furthermore, the genes of the PI3K-Akt signaling pathway in root-knot nematodes were found to be overexpressed following compound treatment [58]. In the current investigation, RT-qPCR analysis revealed that following treatment of *M. enterolobii* with sec-butyl isothiocyanate and geraniol for 12 h and 24 h, the PI3K-Akt-signaling-pathway-related genes (*PIK3R1-1*, *PIK3R1-2*, *Rictor-1*, and *Rictor-2*) were overexpressed. This suggests that the compounds may affect the normal growth and development of the nematode via the PI3K-Akt signaling pathway. However, the specific interaction mechanisms underlying the roles of *PIK3R1* and *Rictor* in root-knot nematodes have not been elucidated, necessitating additional in-depth investigations to clarify this unresolved aspect.

## 4. Materials and Methods

### 4.1. Collection and Preparation of E. japonicum Rhizome Extracts

**Figure 10:**
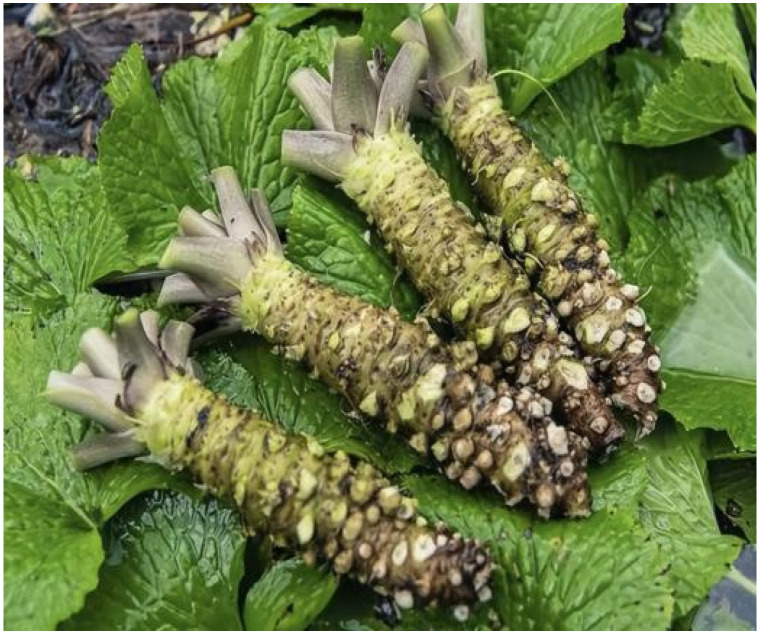
Rhizome of *E. japonicum*.

*Eutrema japonicum* was purchased from Nanjing Yunwei Online Store in July 2023; its place of origin is Baoshan City, Yunnan Province, China (Figure 10). Variety identification was conducted based on the morphological characteristics of the plants: it has dark green leaves with purple at the base, a spreading growth pattern of the plant, and pale green rhizomes, which is consistent with the typical morphological characteristics of *Wasabia japonica* cv. Mazuma [59]. The *E. japonicum* rhizomes were thoroughly cleaned with distilled water to remove soil and surface impurities. The cleaned rhizomes were then sliced using a knife and dried in an oven at a constant temperature of 60 °C until a constant mass was achieved. The dried rhizomes were crushed using a wall-breaking machine (JXFSTPRP-24 grinder, Jingxin Technology Co., Ltd., Shanghai, China) and sieved through a 50-mesh sieve. A 10 g sample of the dried *E. japonicum* powder was placed in a 500 mL Erlenmeyer flask, and 100 mL of 85% ethanol solution was added to achieve a 1:10 ratio. The mixture was extracted by ultrasonication (YM-080S, Fangao Microelectronics Co., Ltd., Shenzhen, China) at 35 °C and 80 W for 2 h. After filtration, the filtrate was evaporated using a rotary evaporator (Heidelberg, Schwarzbach, Germany) at 40 °C for 35 min to form an extractum paste, which was then stored in the refrigerator at 4 °C for later use.

### 4.2. Nematodes

*Meloidogyne enterolobii* were collected from Lingkou Village, Lingkou Town, Ding’an County, Hainan Province, China (N: 19.345925 E: 110.310199); single eggs were purified and preserved in the laboratory [60].

### 4.3. Comparison Test on Co-Solvents for Plant Extracts

The ethanol extract of *E. japonicum* rhizome was obtained as an infusion following rotary evaporation. To solubilize the extract, acetone and DMSO were used as solvents. Specifically, 1 mg of extract was weighed, and 1 mL of acetone or DMSO was added to a 35 mm culture dish. Each treatment was performed in triplicate, and the occurrence of precipitation in the extract was observed after 24 h and 48 h of treatment. In a 12-well cell culture plate, nematode suspension (100 nematodes) was added to each well. Subsequently, 500 μL of 2% aqueous acetone or DMSO was added, and sterile water was used to make up the volume to 1 mL. Sterile water was utilized as a blank control. Nematode mortality was observed and recorded after incubation in a constant temperature chamber at 25 °C for 24 h and 48 h. The mortality rate was calculated following the method described by Wang [15].

### 4.4. Determination of Nematicidal Activity

Using the impregnation method, a nematode suspension (100 nematodes) was added to each well of a 24-well cell culture plate containing 500 μL of of *E. japonicum* rhizome extract solution. The volume was then adjusted to 1 mL with sterile water. After multiple experiments, the sample concentrations were finally determined to be 200, 100, 50, 25, and 12.5 mg/mL, with sterile water as the negative control, 90% abamectin original powder as the chemical control, and 1% aqueous DMSO as the solvent control. Each concentration was repeated three times. The treated cell culture plates were incubated at 25 °C for 24 h and 48 h. Subsequently, the treated nematodes were transferred to centrifuge tubes, centrifuged, and washed twice with sterile water. The nematodes were then transferred to a 3 cm Petri dish, resuscitated by adding sterile water, and left for 48 h. After 48 h of recovery, the nematodes were observed under a microscope. The needle prick test was used to confirm death, as the nematodes remained immobile upon stimulation [15]. The nematode mortality rate and corrected mortality rate were then calculated using Equations (1) and (2):

Mortality rate = Number of dead nematodes/Number of nematodes in the treatment × 100%(1)

Corrected mortality rate = (treatment nematode mortality rate − control nematode mortality rate)/ (1 − control nematode mortality rate) × 100%(2)

### 4.5. Effect of Rhizome of E. japonicum Extract on Hatching of Single Egg of M. enterolobii

Fresh, plump, and yellow-brown egg sacs were carefully selected for incubation experiments. The egg sacs underwent a series of steps: First, they were immersed in a 1.0% sodium hypochlorite solution and agitated for 1 min, followed by centrifugation. The supernatant was discarded, and the bottom solution was washed three times with sterilized water. Subsequently, water was added, and the mixture was shaken to collect the supernatant, resulting in a single-egg suspension [61,62]. Each well of a 12-well cell culture plate received a single-egg suspension (approximately 100 eggs/well), followed by the addition of 500 µL of *E. japonicum* rhizome extract solution to each well. Sterile water was then used to adjust the volume in each well to 1 mL, and final *E. japonicum* rhizome extract solution concentrations of 200, 100, 50, 25, or 12.5 mg/mL were achieved. Sterile water was utilized as a control, and each treatment was replicated three times. Following treatment at 25 °C for durations of 4 h, 8 h, 16 h, 24 h, and 48 h, the extraction solution adhering to the egg surface was centrifuged and rinsed with sterile water. Subsequently, the eggs were transferred to sterile water for further incubation at 25 °C in an incubator. After four days, egg hatching was observed under a stereomicroscope, and the hatching inhibition rate was calculated following the method described by Qiu [62] using Equations (3) and (4):

Hatching rate = Total number of incubations/Total number of eggs × 100%(3)

Incubation inhibition rate = Control hatching rate − treatment hatching rate/Control hatching rate × 100%(4)

### 4.6. Analysis of the Chemical Composition of E. japonicum Rhizome Extract

#### 4.6.1. GC-MS-VOCs Analysis

To elucidate the active compounds present in the ethanol extract of *E. japonicum* rhizome, the active substances were identified using Gas Chromatography–Mass Spectrometry (GC-MS) for Volatile Organic Compounds (VOCs) analysis. The solid-phase microextraction (SPME) cycle of the PAL rail system comprised the following steps: incubation temperature set at 60 °C; preheat for 15 min; incubate for 30 min; and desorption for 4 min. The GC-MS analysis was conducted using an Agilent 7890 gas chromatograph system connected to a 5977B (Agilent, Santa Clara, CA, USA) mass spectrometer (Lico, San Jose, CA, USA). The system employed DB-Wax as the medium, with injection performed in splitless mode. Helium served as the carrier gas, with a front inlet purge flow of 3 mL/min and a gas flow rate through the column of 1 mL/min. The initial temperature was maintained at 40 °C for 4 min, then ramped up to 245 °C at a rate of 5 °C/min and held at steady temperature for 5 min. The temperatures of the injection port, transfer line, ion source, and quadrupole were set at 250, 250, 230, and 150 °C, respectively. The electron impact mode was used with an electron energy of 70 eV. Mass spectrometry data were collected in scan mode within an *m*/*z* range of 20–400, with a solvent delay of 2.13 min.

#### 4.6.2. Qualitative Analysis Was Conducted on Chemical Composition of *E. japonicum*

ChromaTOF software (Version 4.3x, LECO Corporation, Saint Joseph, MI, USA) was used for peak extraction, baseline correction, deconvolution, peak integration, peak alignment, and mass spectrometry matching analysis of mass spectrometry data [63]. The mass spectral and retention time indices were matched with the compounds in the LECO-Fiehn Rtx5 database, followed by comparison with databases such as KEGG and HMDB as well as a self-constructed database from Shanghai Biotree Biomedical Technology (Shanghai, China).

### 4.7. Preparation of Sec-Butyl Isothiocyanate and Geraniol Solution

Pure chemical sec-butyl isothiocyanate (CAS number: 4426-79-3) and pure chemical geraniol (CAS number: 106-24-1) were purchased from Aladdin Reagent Shanghai (≥98% purity). DMSO was used as the solvent to prepare the stock solutions of sec-butyl isothiocyanate and geraniol, both with a concentration of 100 mg/mL. Subsequently, the above two stock solutions were gradually diluted with DMSO to reduce their concentrations to 1 mg/mL. Finally, the diluted solutions were further diluted with sterile water to the working concentrations required for the experiment, and the volume fraction of DMSO in the working solutions was controlled to be ≤0.5%.

### 4.8. Determination of the Activity of Sec-Butyl Isothiocyanate from E. japonicum Extract Against J2s and Single-Egg Hatching of M. enterolobii

#### 4.8.1. Effect of Sec-Butyl Isothiocyanate on J2s of *M. enterolobii*

Using the test method described in Section 4.5, 500 µL nematode suspension (100 nematodes) was added to each well of a 24-well cell culture plate containing 500 µL of sec-butyl isothiocyanate solution in each well. Final sec-butyl isothiocyanate solution concentrations were 10, 5, 2.5, 1.25, or 0.625 µg/mL. We calculated and recorded the mortality rate and corrected mortality rate after 24 and 48 h.

#### 4.8.2. Effect of Sec-Butyl Isothiocyanate on Single-Egg Hatching

Using the test method described in Section 4.6, each well of a 12-well cell culture plate received 500 µL single-egg suspension (100 particles/well), followed by the addition of 500 µL of sec-butyl isothiocyanate solution to each well. Final sec-butyl isothiocyanate solution concentrations were 10, 5, 2.5, 1.25, or 0.625 µg/mL. We calculated and recorded the single-egg hatching rate and incubation inhibition rate after 4, 8, 16, 24, and 48 h.

### 4.9. Determination of the Activity of Geraniol from E. japonicum Extract Against J2s and Single-Egg Hatching of M. enterolobii

#### 4.9.1. Effect of Geraniol on J2s of *M. enterolobii*

Using the test method described in Section 4.5, 500 µL nematode suspension (100 nematodes) was added to each well of a 24-well cell culture plate containing 500 µL of geraniol solution in each well. Final geraniol solution concentrations were 5, 2.5, 1.25, 0.625, or 0.3125 μg/mL. We calculated and recorded the mortality rate and corrected mortality rate after 24 and 48 h.

#### 4.9.2. Effect of Geraniol on the Hatching of Single Egg of *M. enterolobii*

Using the test method described in Section 4.6, each well of a 12-well cell culture plate received 500 µL single-egg suspension (100 particles/well), followed by the addition of 500 μL of geraniol solution to each well. Final geraniol solution concentrations were 5, 2.5, 1.25, 0.625, or 0.3125 μg/mL. We calculated and recorded the single-egg hatching rate and incubation inhibition rate after 4, 8, 16, 24, and 48 h.

### 4.10. Network Pharmacology Analysis

To gain further insights into the potential mechanisms of the inhibitory effect of the active compounds in *E. japonicum* on *M. enterolobii*, the network pharmacology approach was employed to predict the relevant targets of these compounds through the following steps:

1. Compound screening: On the basis of the previously described GC-MS-VOCs analysis, chemical composition data of the *E. japonicum* extract were acquired. Subsequently, 10 active compounds were initially screened via literature investigation.
2. Acquisition of potential targets for active compounds: Potential targets of 10 components were queried via databases commonly used in network pharmacology research (TCMSP, TCMID, and BATMAN-TCM). The SMILES sequences corresponding to these potential targets were downloaded from the PubChem database and input into the Swiss Target Prediction database, and all target information was consolidated with duplicate values removed to ultimately obtain the targets corresponding to each compound.
3. Targets acquisition for root-knot nematode: Based on the information from the academic databases GeneCards [https://www.genecards.org/ (accessed on 14 January 2025)], OMIM [https://omim.org/ (accessed on 14 January 2025)], TTD [http://db.idrblab.net/ttd/ (accessed on 14 January 2025)], CTD [http://ctdbase.org/ (accessed on 14 January 2025)], DisGeNET [https://www.disgenet.org/ (accessed on 14 January 2025)], and NCBI Gene [https://www.ncbi.nlm.nih.gov/gene/ (accessed on 14 January 2025)], potential targets of root-knot nematodes were screened. Subsequently, all screened targets were merged, duplicates entries were removed, and the final targets corresponding to root-knot nematode were identified.
4. Screening of common targets and construction of protein–protein interaction (PPI) network: By screening the common targets of active compounds and root-knot nematodes, we identified these shared targets as potential action sites of *E. japonicum* active compounds on root-knot nematodes. The STRING database [https://string-db.org (accessed on 19 February 2025)] was used to construct a PPI network, and Cytoscape software version 3.10.2 was used to build a compound–target network; nodes with higher degree values were selected as candidate targets.
5. GO and KEGG analysis: The Metascape database version 3.5.20241201 [https://metascape.org/gp/index.html#/main/step1 (accessed on 19 February 2025)] was used for GO and KEGG pathway enrichment analysis.
6. Construction of the compound–target protein interaction network: The names of 10 compounds and corresponding targets from 20 KEGG pathways (with the lowest *p*-values in enrichment analysis) were imported into Cytoscape to construct the compound–target protein interaction network.

### 4.11. Real-Time Quantitative PCR (RT-qPCR) Analysis

Based on the LC_50_ values from Section 4.8.1 and Section 4.9.1, the immersion method was employed: 375 µL of sec-butyl isothiocyanate solution was added to each well of a 24-well cell culture plate along with a J2 suspension (approximately 7000 nematodes), followed by the addition of sterile water to bring the volume to 3 mL. The final concentration of sec-butyl isothiocyanate solution was 1.25 μg/mL. Meanwhile, sterile water was used to treat nematodes as the control group. At 0 h, 12 h, 24 h, and 36 h post-treatment, nematodes were collected in 1.5 mL centrifuge tubes at a centrifugation speed of 12,000 rpm. After aspirating the supernatant, the nematodes were washed with sterile water, then flash-frozen in liquid nitrogen and stored at −80 °C. Each treatment had three biological replicates. The above steps were repeated to collect nematode samples treated with geraniol for 0 h, 12 h, 24 h, and 36 h.

Total RNA from nematodes was isolated using the Total RNA Extraction Kit for Trace Samples (TIANGEN, DP420, Tiangen Biotech Co., Ltd., Beijing, China). cDNA was synthesized with the Primer Reverse Transcription Kit (Thermo Scientific, M1681, Thermo Fisher Scientific Inc., Shanghai, China). RT-qPCR primers were designed using Snap Gene software (version 6.0.2) (Table S4), and primers were synthesized by Sangon Biotech (Shanghai, China). RT-qPCR was performed using SYBR Premix Ex Taq (Vazyme, Q711-02), with the *Actin* gene of *M. enterolobii* as the reference gene. All RT-qPCR assays were conducted in triplicate.

### 4.12. Statistical Analysis

#### 4.12.1. Data Processing for Root-Knot Nematode Bioactivity Assay

Following the method of Xiaoli Dou (2024) [60], we performed data statistics, variance analysis, and visualization.

#### 4.12.2. Qualitative Analysis of the Chemical Composition of *E. japonicum* Rhizome Extract

Following the method of Xiaoli Dou (2024) [60], we performed peak extraction, analyzed the mass spectral data, and compared the compounds data.

#### 4.12.3. Data Analysis and Visualization for Network Pharmacology Analysis

The target protein data were summarized using Excel. The Venn diagram was created using the Venny software version 2.1 [https://bioinfogp.cnb.csic.es/tools/venny/index.html (accessed on 29 September 2025)], the PPI network was constructed using the STRING online software [http://string-db.org (accessed on 19 February 2025)], the GO and KEGG enrichment analysis graphs were created using the SRplot online software version 2021–2025 [http://bioinformatics.com.cn/ (accessed on 19 February 2025)], and the composite target network was constructed using Cytoscape software version 3.10.2.

#### 4.12.4. Data Analysis and Visualization for RT-qPCR Analysis

Data statistics were performed using Excel, one-way analysis of variance (ANOVA) was performed using IBM SPSS Statistics 25, and statistical significance was defined as *p* < 0.05. Data visualization was conducted using Origin 2018.

## Figures and Tables

**Table 1:** Toxic effects of different co-solvent against second-stage juveniles (J2s) of *M. enterolobii*.

Treatment	Precipitation (24 h)	Mean Mortality Rate (%) ± SEM (24 h)	Mean Mortality Rate (%) ± SEM (48 h)
DMSO	No Precipitate	0.00	0.00
Acetone	Precipitate	5.333 ± 0.578	10.333 ± 1.528
Sterile water	Precipitate	0.00	0.00

SEM = standard error of the mean.

**Table 2:** Toxicity of different treatments against second-stage juveniles (J2s) of *M. enterolobii*.

Treatment	Concentration (mg/mL)	Mean Mortality Rate (%) ± SEM (24 h)	Mean Mortality Rate (%) ± SEM (48 h)
*E. japonicum* rhizome extract	200	71.81 ± 2.082 a	88.93 ± 2.082 a
	100	60.06 ± 1.453 b	72.49 ± 1.453 b
	50	47.65 ± 1.528 c	59.06 ± 2.333 c
	25	42.28 ± 1.453 d	50.67 ± 2.082 d
	12.5	33.89 ± 2.33 e	42.28 ± 1.453 e
90% Abamectin original powder	0.1	63.33 ± 1.527 b	68.33 ± 1.527 b
DMSO	—	1.33 ± 0.577	1.33 ± 0.577
Sterile water	—	0.00	0.00

Columns marked with the different letters indicate significant differences (*p* < 0.05) as tested by Tukey’s method. SEM = standard error of the mean.

**Table 3:** Toxicity of *E. japonicum* rhizome extract on *M. enterolobii*.

Extract	Period of Treatment (h)	Linear Equation y = ax + b	LC_50_ (mg/mL)	LC_90_ (mg/mL)	Correlation Coefficient (R)
*E. japonicum* rhizome extract	24	y = 0.189x + 36.847	69.59	281.23	0.916
	48	y = 0.235x + 44.751	22.336	192.55	0.940

LC = lethal concentration.

**Table 4:** GC-MS-VOCs analysis of some chemical constituents of the ethanol extract from the *E. japonicum* rhizome extract.

No.	Compound Name	Molecular Formula	Biotree Class	Relative Content (%)	Chemical Structure	Reference
1	2-Butenal, (E)-	C_4_H_6_O	Organooxygen compounds	0.086	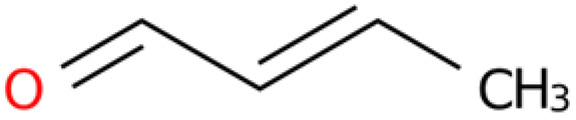	[27]
2	Butane,1-isothiocyanato-	C_5_H_9_NS	Isothiocyanates	0.0423	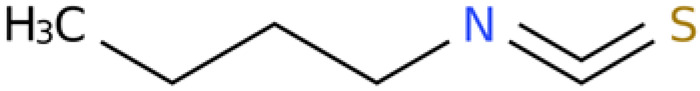	[28,29,30]
3	Butane,2-isothiocyanato-	C_5_H_9_NS	Isothiocyanates	5.629	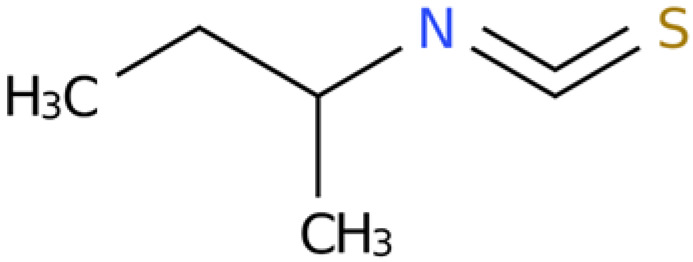	[28,29,30]
4	Acetaldehyde,tetramer	C_8_H_16_O_4_	Organooxygen compounds	3.184	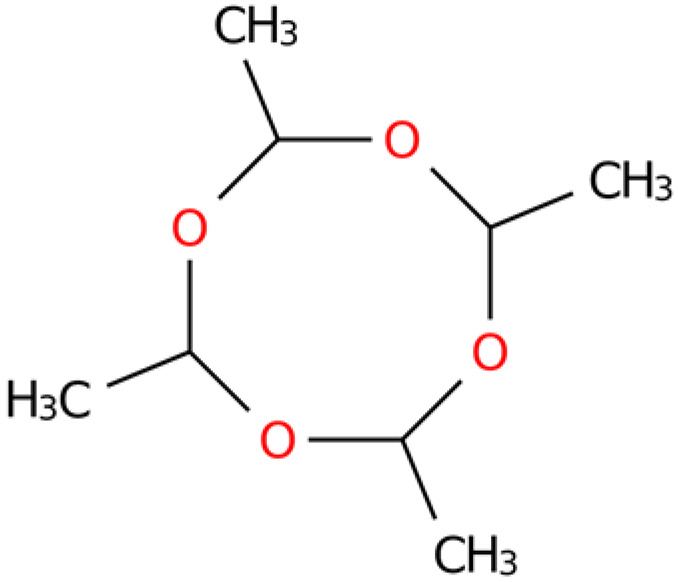	[27]
5	Furan,tetrahydro-2,2,4,4-tetramethyl-	C_8_H_16_O	Tetrahydrofurans	0.6638	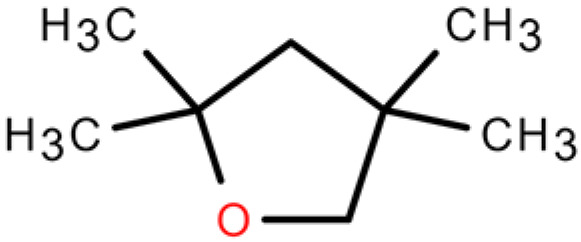	[31]
6	Geraniol	C_10_H_18_O	Prenol lipids	0.387	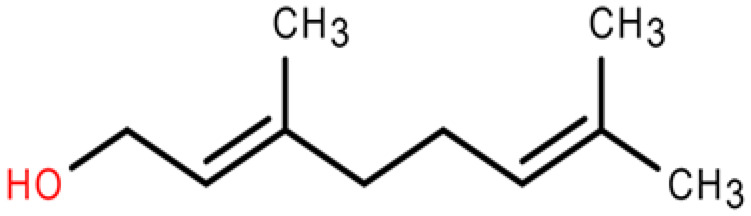	[32]
7	Isopropyl isothiocyanate	C_4_H_7_NS	Isothiocyanates	1.041	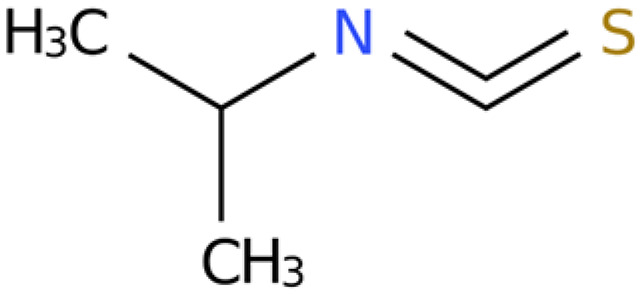	[28,29,30]
8	2(4H)-Benzofuranone, 5,6,7,7a-tetrahydro-4,4,7a-trimethyl-,(R)-	C_11_H_16_O_2_	Benzofurans	0.185	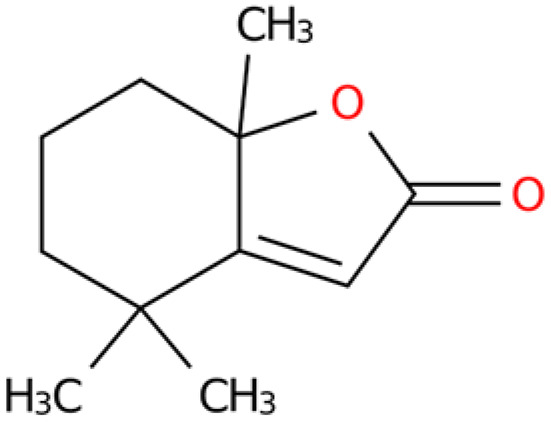	[33]
9	2(5H)-Furanone	C_4_H_4_O_2_	Dihydrofurans	0.1781	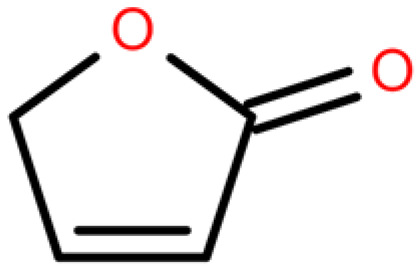	[33]
10	Pyrazine, tetramethyl-	C_8_H_12_N_2_	Diazines	0.8013	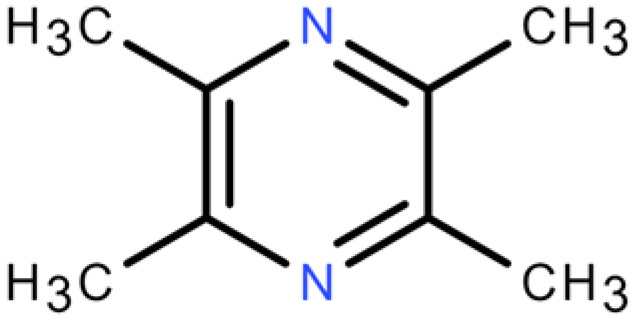	[34]

**Table 5:** Toxicity of different treatments against second-stage juveniles (J2s) of *M. enterolobii*.

Treatment	Concentration (µg/mL)	Mean Mortality Rate (%) ± SEM (24 h)	Mean Mortality Rate (%) ± SEM (48 h)
sec-butyl isothiocyanate	10	92.62 ± 2.5166 a	100 ± 0.00 a
	5	72.49 ± 2.5166 b	82.55 ± 2.5166 b
	2.5	65.10 ± 2.5166 c	70.13 ± 1.5275 c
	1.25	57.38 ± 2.5166 d	62.67 ± 1.5275 d
	0.625	44.96 ± 2.5166 e	51 ± 1.1547 e
90% Abamectin original powder	100	63.33 ± 1.527 c	67.9 ± 1.53 c
Sterile water	—	0.00	0.00

Columns marked with the different letters indicate significant differences (*p* < 0.05) as tested by Tukey’s method. SEM = standard error of the mean.

**Table 6:** Analysis of toxicity results of sec-butyl isothiocyanate against *M. enterolobii*.

Extract	Period of Treatment	Linear Equation y = ax + b	LC_50_ (µg/mL)	LC_90_ (µg/mL)	Correlation Coefficient
sec-butyl isothiocyanate	24 h	y = 3.811x + 45.855	1.087	9.06	0.915
	48 h	y = 4.302x + 49.827	0.04	9.338	0.931

LC = lethal concentration.

**Table 7:** Toxicity of different treatments against second-stage juveniles (J2s) of *M. enterolobii*.

Treatment	Concentration (µg/mL)	Mean Mortality Rate (%) ± SEM (24 h)	Mean Mortality Rate (%) ± SEM (48 h)
geraniol	5	85.57 ± 3.05505 a	100 ± 0.00 a
	2.5	70.13 ± 1.52753 b	82.21 ± 2.08167 b
	1.25	62.01 ± 2.51661 c	70.80 ± 1.73205 c
	0.625	50.33 ± 1.52753 d	58.05 ± 1.52753 e
	0.3125	43.29 ± 1.52753 e	50.34 ± 1.15470 f
90% Abamectin original powder	100	63.33 ± 1.527 c	68.33 ± 1.527 d
Sterile water	—	0.00	0.00

Columns marked with the different letters indicate significant differences (*p* < 0.05) as tested by Tukey’s method. SEM = standard error of the mean.

**Table 8:** Analysis of toxicity results of geraniol against *M. enterolobii*.

Extract	Period of Treatment	Linear Equation y = ax + b	LC_50_ (µg/mL)	LC_90_ (µg/mL)	Correlation Coefficient
geraniol	24 h	y = 7.081x + 42.792	1.018	6.67	0.908
	48 h	y = 8.543x + 49.474	0.062	4.744	0.923

LC = lethal concentration.

## Data Availability

Data from this study are depicted in the figures and tables included in this article.

## Supplementary Materials

The following supporting information can be downloaded at: https://www.mdpi.com/article/10.3390/plants14213310/s1, Table S1: Potential targets of active compounds; Table S2: Target collection of root-knot nematode; Table S3: PIK3R1 and mTOR proteins information; Table S4: RT-qPCR primer sequences.
